# Microsclerotia formation of the biocontrol fungus *Cordyceps javanica* IF-1106 and evaluation of its stress tolerance and pathogenicity

**DOI:** 10.3389/fmicb.2025.1583850

**Published:** 2025-04-29

**Authors:** Yihua Li, Junmei Li, Xiaoxia Cai, Meiyu Gao, Hongliang Diao, Huiming Xiang, Wenwen Zhou, Ruiyan Ma

**Affiliations:** College of Plant Protection, Shanxi Agricultural University, Jinzhong, China

**Keywords:** entomopathogenic fungi, *Cordyceps javanica*, microsclerotia, cell surface hydrophobicity, environmental persistence, pathogenicity

## Abstract

**Introduction:**

*Cordyceps javanica* IF-1106 is an entomopathogenic fungus with a wide range of hosts. It produces microsclerotia in liquid media.

**Methods:**

This study took a close observation of the microsclerotia formation process of *C. javanica* IF-1106 and described the surface characteristics of *C. javanica* IF-1106 microsclerotia. Secondly, the viability of microsclerotia under high temperature and ultraviolet radiation were determined. Thirdly, the microsclerotia were stored under different temperatures to assess storage stability. Finally, activity of microsclerotia against root-knot nematode and the influence on the plant growth of cucumber were investigated.

**Results and discussion:**

Spores germinate quickly, and hyphal elements clump together, forming hydrophobic microsclerotia. The microsclerotia have 100% survival under 55°C and UV-B radiation of 3 J cm^−2^. Following such exposure, the microsclerotia are able to germinate and produce conidia. After 1 year of storage at either room temperature (25°C) or refrigeration (−80°C, −20°C, and 4°C), all the microsclerotia germinated and spore yield was more than 4 × 10^9^ spores g^−1^. Finally, *C. javanica* IF-1106 microsclerotia showed ovicidal activity against root-knot nematode eggs, and a glasshouse pot trial indicated that control efficiency of microsclerotia against root-knot nematodes reached 92.6%, which improved the growth of the test cucumber plants. These attributes suggest that microsclerotia of this fungus can be applied as a biocontrol agent to control soil borne pest nematodes.

## Highlights

- Microsclerotia of *Cordyceps javanica* IF-1106 had higher hydrophobicity than other cell types.
- Microsclerotia of *C. javanica* had 100% survival after treated at 55°C for 3 h or by UV-B radiation for 3 J cm^−2^.
- Microsclerotia had 100% viability after 1 year of storage at 25°C.
- Microsclerotia showed high ovicidal activity against root-knot nematode and had a positive impact on cucumber growth.

## 1 Introduction

Concerns regarding the ecological and health risks of exposure to synthetic insecticides have stimulated the search for alternative methods for controlling crop pests (Liu et al., 2023). Entomopathogenic fungi are well-documented biological control agents that have great potential for controlling agricultural insect pests worldwide (Sharma et al., 2023). Among the successful applications of such fungi are the use of species of *Beauveria*, *Metarhizium*, *Lecanicillium*, *Cordyceps* against a variety of economically important arthropod pests such as locusts and grasshoppers, soil dwelling insects, piercing and sucking insects, mites, stored-grain pests, several forestry pests, as well as some medical and veterinary pests (Quesada-Moraga et al., 2023).

Among these fungi, *Cordyceps javanica* (Hypocreales: Cordycipitaceae) has been considered for pest control of pests such as aphids and whiteflies, and it has great potential for control of a variety of additional arthropod species (Wang et al., 2023; Dimase et al., 2024). Different infectious *C. javanica* propagules can be produced and applied for pest control. Thus, in addition to mycelial and hyphal growth forms, *C. javanica* produces a number of mono-nucleated single cell types, including aerial conidia, blastospores, submerged conidia, and budding hyphal cells from the developing hyphae (micro-cycle conidiation) (Rivas-Franco et al., 2020; Gotti et al., 2023). Conidia are reproductive structures critical for fungal distribution and survival, and the environment conditions experienced during conidiation can affect the properties of conidia, such as their morphology, germination, and pathogenicity (Hagiwara et al., 2017). Blastospores are thin-walled cells resembling yeast that are less tolerant to conditions such as high temperature, UV radiation, and desiccation, which severely limits their use in pest management (Mascarin et al., 2021; das Chagas Bernardo et al., 2020; Li et al., 2024). For example, exposure to high temperatures or solar radiation (particularly UV-B light) for a few hours is sufficient to inactivate most *Beauveria bassiana* conidia, inhibit spore germination, and reduce their virulence (Quesada-Moraga et al., 2023; Ying and Feng, 2011).

In addition to the above stages, some entomopathogenic fungi produce overwintering dormant structures known as microsclerotia, which produce conidia when rehydrated (de Lira et al., 2020). Microsclerotia have been described as a compact, melanized, hyphal structure (Villamizar et al., 2018). These structures offer many advantages in pest control due to the formation of infective conidia *in situ* and their persistence in environments with adverse conditions (Song et al., 2014). Microsclerotia of some fungal species have been developed as biocontrol agents, including in such species as *Metarhizium humberi*, *M. brunneum*, and *B. bassiana* (Rodrigues et al., 2021; Goble et al., 2016; Zhang et al., 2019). Recent studies have shown that *M. robertsii* and *M. brunneum* F52 to produce desiccation-tolerant microsclerotia granules able to control the tick *Rhipicephalus microplus*, the wood borer, *Anoplophora glabripennis*, and the annual bluegrass weevil, *Listronotus maculicollis* (Marciano et al., 2021; Goble et al., 2015; Koppenhӧfer et al., 2020). Microsclerotia of *B. bassiana* are stable under different storage conditions up to 1 year and retained high germination even under thermal or UV-B radiation (1,200 mWm^−2^) stress for 72 h (Yousef-Yousef et al., 2022). However, the application of microsclerotia has not reached its full potential due to our incomplete understanding of the mechanism of their formation, their surface properties, and their interaction with plants and target pests.

The ability to form infective conidia *in situ* makes microsclerotia a particularly suitable agent for control of soil-dwelling pests. Among such soil-borne pests are root-knot nematodes (RKNs), which are a major threat to vegetables, fruits and ornamental plants. RKNs can be suppressed using chemical nematicides, but the use of many such products have recently been restricted due to their impacts on human health and non-target organisms. Microsclerotia of some fungi have shown promise against RKNs (Kepenekci et al., 2017).

Microsclerotia of *Cordyceps* species can be artificially produced using liquid fermentation (Catão et al., 2021). However, many of details concerning formation of microsclerotia in *C. javanica*, including surface characteristics, resistance levels to environmental stresses, and efficacy against particular target pests are unknown.

In this study, we examined the processes leading to the formation of microsclerotia in *C. javanica* IF-1106 and described the surface characteristics of the microsclerotia. Secondly, we determined the viability of *C. javanica* microsclerotia under high temperature and ultraviolet radiation. We also determined the effectiveness of long-term storage of the microsclerotia of this species when held under different temperatures. Finally, we measured the activity of microsclerotia against root-knot nematodes and the influence of their application on the growth of greenhouse cucumber crops.

## 2 Materials and methods

### 2.1 Fungal strains and culture methods

*Cordyceps javanica* (Hypocreales: Cordycipitaceae) IF-1106 was isolated from adults of the whitefly *Bemisia tabaci* biotype B (Hemiptera: Aleyrodidae) collected from Taigu (37°25′26″N, 112°35′36″E), China, in 2011 (Li et al., 2022). The fungus was stored in 10% glycerol at −80°C in the Insect Ecology and Biocontrol Laboratory, Shanxi Agricultural University. The isolate was cultivated on PDA medium under controlled temperature and relative humidity (25 ± 1°C and RH ≥ 80%).

### 2.2 Production of *Cordyceps javanica* microsclerotia

Fresh conidia from 10-day-old cultures of *C. javanica* were scraped from PDA culture plates and suspended in a 0.1% v/v Tween-80 sterile distilled water solution. Suspensions were filtered through four layers of medical gauze to remove conidial aggregates, and the suspensions were adjusted to 1.0 × 10^7^ conidia mL^−1^.

The medium used for inducing microsclerotia production was as follows (all values are per liter of deionized water): 40.0 g of sucrose (total C = 42%), 5.0 g yeast extract powder (total N ≥ 9%), 10.0 g of MgSO_4_, 10.0 g of KCl, 20.0 g of KH_2_PO_4_, 5.0 × 10^−4^ g MnSO_4_·H_2_O, 5.0 × 10^−4^ g CuSO_4_·5H_2_O, 1.0 × 10^−3^ g FeSO_4_·7H_2_O, 5.0 × 10^−4^ g FeCl_3_·6H_2_O, 5.0 × 10^−4^ g vitamin B_2_, 2.0 × 10^−3^ g vitamin B_5_, 5.0 × 10^−3^ g vitamin B_6_, and 3.0 × 10^−3^ g vitamin B_9_ were combined to produce a medium with a carbon-to-nitrogen ratio (C:N) of 37:1.

The controlled culture was carried out with basic medium to produce hyphae and blastospores, the composition of which was as follows (all values were per liter of deionized water): 40.0 g of sucrose (total C = 42%), 5.0 g soy peptone (total N ≥ 9%), 10.0 g of MgSO_4_, 10.0 g of KCl, 20.0 g of KH_2_PO_4_, and 2.0 g of FeSO_4_·7H_2_O, which also produced a medium with a carbon-to-nitrogen ratio (C:N) of 37:1. All chemicals were analytically pure.

Three flasks of media were inoculated with a 1.0 × 10^7^ conidia mL^−1^ suspension of the fungal strain at 25°C. The flasks were then shaken at 180 rpm in a rotary shaker incubator for 7 days for microsclerotia production.

### 2.3 Micromorphological observations of microsclerotia

To monitor the formation of *C. javanica* microsclerotia, samples were collected from fermentation flasks at 12, 24, 36, 48, 60, and 72 h for examination under an optical microscope. Samples for examination with a scanning electron microscope (SEM) were prepared and viewed as described by Wang et al. (2023). SEM samples were first immersed in 2.5% v/v glutaraldehyde (Macklin, Shanghai, China) overnight, and then rinsed three times with 0.1 M phosphoric acid buffer (pH 7.2, 10 min each time). Next, samples were dehydrated with 30, 50, 70, 80, and 95% ethanol by rinsing (10 min per grade). The final dehydrated samples were then treated with 100% ethanol twice. The samples were mounted on a microscope slide and sputter-coated with gold using auto fine coater (MC-1000, HITACHI). The samples were observed by SEM (JSM-6490LV, JEOL, Japan).

### 2.4 Determination of cell surface hydrophobicity

Fresh aerial conidia were harvested from 10-day-old cultures of *Cordyceps javanica* grown on PDA plates by gently scraping the surface with PUM buffer. Blastospores and hyphae were obtained from basic media (2.2) and subsequently separated using sterile gauze. The blastospores, present in the filtrate, were collected by centrifugation at 4,472 × g for 15 min. Hyphae retained on the sterile gauze were washed with PUM buffer. Additionally, microsclerotia were collected from induction media (2.2) using sterile gauze for further processing. Aerial conidia, blastospores, hyphae, or microsclerotia were washed in PUM buffer (per liter: 22.2 g K_2_HPO_4_, 7.26 g KH_2_PO_4_, 1.8 g urea, 0.2 g MgSO_4_·7H_2_O, final pH 7.1). Hexadecane (1 mL) was then added to each tube and the tubes were vortexed three times for 30 s. The vortexed tubes were allowed to stand at room temperature for 15 min. Cell surface hydrophobicity was determined using the microbial adhesion to hydrocarbons (MATH) assay as described by Holder et al. (2007).

### 2.5 Contact angle determination

Contact angle measurements of the *C. javanica* cell types were determined using JC2000D1 software with automated drop dispenser and tilting plate. Increasing angle measurements were made just before movement of the water droplet. Briefly, a drop of solution was placed onto the surface of the substrate to be tested. The leading edge (dynamic) contact angle was then determined. Experiments were performed with sterile dH_2_O at room temperature and 50–55% RH. Each experiment was performed using at least 10 drops from each of three samples.

### 2.6 Thermotolerance of *Cordyceps javanica* microsclerotia

Fresh batches of *C. javanica* microsclerotia were produced following the methodology described above. Microsclerotia were collected from growth media 6 days after initiation by adding 5 g of diatomaceous earth (DE) to each 100 mL of culture broth. The broth was then vacuum-filtered in a Buchner funnel through Whatman No. 1 filter paper to remove spent media. The resulting filter cakes were layered on glass Petri plates (15 cm diameter) and air-dried overnight with a laminar-flow and until the moisture level of the cakes was 3–5%. These cakes were formulations of microsclerotia in diatomaceous earth (“microsclerotia-DE formulation”) (Rivas-Franco et al., 2020), which was the source of the microsclerotia used in our experiments.

The microsclerotia-DE formulation of 25 mg was placed into a plastic centrifuge tube and held for 0, 0.5, 1.0, 1.5, 2.0, 2.5, or 3.0 h, at 40, 45, 50, or 55°C. The formulated microsclerotia were then used to inoculate a water-agar plate medium. These plates were then cultured for 24 h at 25°C to determine the rate of germination and spore yield under an optical microscope. A fresh conidial spore suspension (1 × 10^7^ spores mL^−1^) was used as the control. Conidia were considered germinated when germ tubes measured at least conidial diameter (Mascarin et al., 2018). The presence or absence of hyphal growth from each microsclerotia was evaluated with a dissecting microscope. Microsclerotia were considered germinated upon hyphal development around the microsclerotia (Microsclerotia hyphal germination) (Corval et al., 2021). The germination rate was calculated as the percentage of germinated conidia and microsclerotia. Each treatment (storage time × storage temperature) was carried out in triplicate.

### 2.7 UV-B tolerance of *Cordyceps javanica* microsclerotia

A 25 mg sample of the microsclerotia-DE formulation was evenly sprinkled on a water-agar plate (9 cm diameter), and the plate was then placed in an irradiation chamber (SCIENTZ 03-II, SCIENTZ Biotechnology Co., Ltd.) where it was irradiated by a UV lamp (UV-B wavelength: 308 nm). The UV-B radiation treatment levels were 0, 1.0, 1.5, 2.0, 2.5, 3.0 J cm^−2^. Microsclerotia were then moved into a 25°C incubator and held for 24 h after which the plates were examined under an optical microscope to determine the rate of germination of the microsclerotia. A fresh conidial suspension (1 × 10^7^) was used as the control, and each treatment (levels of UV radiation) was carried out in triplicate (Yin and Wang, 2022).

### 2.8 Storage stability of *Cordyceps javanica* microsclerotia

Microsclerotia viability and conidial yield of microsclerotia after long term storage were evaluated using a modification of the method of Jaronski and Jackson (2008). Briefly, the microsclerotia-DE formulation was stored at −80°C, −20°C, and 4°C, or room temperature (25°C) and evaluated after 12 months. The stored microsclerotia-DE formulation from each treatment was sprinkled onto the surface of a water agar plate, which was then incubated for 7 days at 25°C. The conidial suspension on each plate was then sampled by pipetting a portion from each individual rinsed plate, and we determined the number of spores g^−1^ microsclerotia-DE using a hemacytometer (Behle and Jackson, 2014).

### 2.9 Ovicidal activity against root-knot nematode of *Cordyceps javanica* IF-1106 microsclerotia

Root-knot nematodes were purchased from the Jidun Biological Technology Co., Ltd., Zhejiang, China. The concentration of nematode eggs in a suspension was adjusted to 200 eggs mL^−1^. A 12-well tissue culture plate was used for the test, and we added 1 mL of microsclerotia suspension and 1 mL of nematode egg suspension to each well Fungal and nematode egg solutions were evenly mixed and cultured at 25°C for 7 days. Five microsclerotia concentrations (50, 100, 500, 1,000, and 2,000 microsclerotia mL^−1^) were tested, and sterile water was used as the control. The parasitism and hatch ratio of the nematode eggs were calculated. Each treatment (five fungal concentrations) was replicated five times.

### 2.10 Glasshouse pot trial

#### 2.10.1 Preparation of microsclerotia and cucumber seedlings

The microsclerotia fermentation liquid for the formulation used to treat cucumber seedling was adjusted to 500 microsclerotia pieces mL^−1^. Diatomaceous earth and microsclerotia fermentation liquid were then mixed at the ratio of 1:20 (g mL^−1^) for adsorption. The product was then dried in the shade (Yuan et al., 2022).

Cucumber’s seeds (“JingpinChunsi”, purchased from Shandong Lu Vegetable Seed Industry Co., Ltd., Jinan, Shandong) were disinfected with 2% NaClO for 5 min, washed with sterile water 3 times, and seeded into a seedling tray (54 cm × 28 cm, 5 × 10 planting holes).

#### 2.10.2 Preparation of RKN-contaminated soil

Field soil was sieved to remove soil particles larger than 1 cm^3^, then mixed with potting soil at a ratio of 1: 1, and autoclaved for 30 min at 115°C. After the soil cooled down to room temperature, the nematode egg suspension was added into the soil and mixed thoroughly, so that the nematode level in the soil was 200 eggs/100 g soil.

Microsclerotia were mixed with RKN-inoculated soil at one of five concentrations (50, 100, 500, 1,000, and 2,000 pieces/100 g soil). Positive controls included 1 × 10^7^ fungal conidia/100 g soil and water. Cucumber seedlings with 2 to 3 main leaves were transplanted into RKN-inoculated soil. There was one plant per pot. Plant height (cm), fresh weight (g) of above ground plant tissues, root length (cm) (Qiao et al., 2024), and number of root knots were measured to determine control efficiency (Yuan et al., 2022).

### 2.11 Statistical analysis

Treatment significance was determined with ANOVA, analysis, following by mean separation with Duncan’s multiple range tests at *p* < 0.05. SPSS (IBM Corporation 2017) and Origin (Origin Lab Corporation 2021) programs were used to analyze data and construct graphs.

## 3 Results

### 3.1 Microsclerotia development

**Figure 1:**
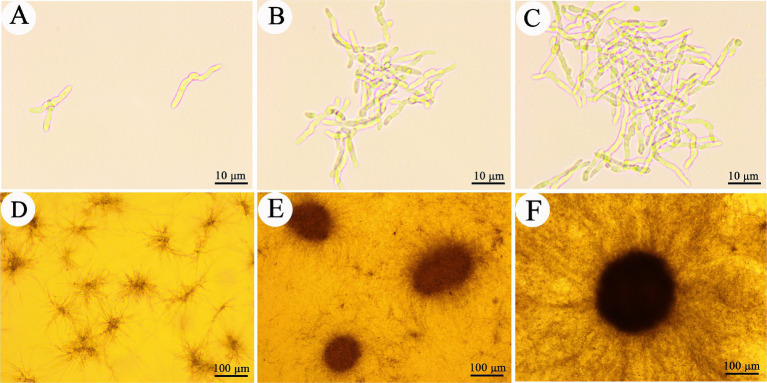
Development of *Cordyceps javanica* IF-1106 microsclerotia in liquid cultures. **(A–C)** 12 h, conidia germinated and developed to hypha. **(D)** 24 h, hyphae begin to aggregate. **(E)** 36 h, microsclerotia begin to form. **(F)** Well developed microsclerotia.

Microsclerotia are compact, melanized aggregates that have been recognized in fungi as overwintering structures. *Cordyceps javanica* IF-1106 was able to form microsclerotia in liquid media (Figure 1). When conidia are inoculated into liquid media, they germinate and initially dispersive hyphae within the first 12 h. Also, in the first 12 h, we observed early stages of hyphae aggregation (Figures 1A–C). By 24 h, hypha showed strong aggregation and microsclerotia formation, although the structure of the microsclerotia was relatively loose (Figure 1D). From 24 to 36 h, compact and strongly melanized microsclerotia were observed (Figures 1E,F). The core diameter of these microsclerotium structures ranged from 50–200 μm, while the peripheral mycelium ranged from 100–300 μm from the edge of the compact microsclerotia (Figure 1F).

**Figure 2:**
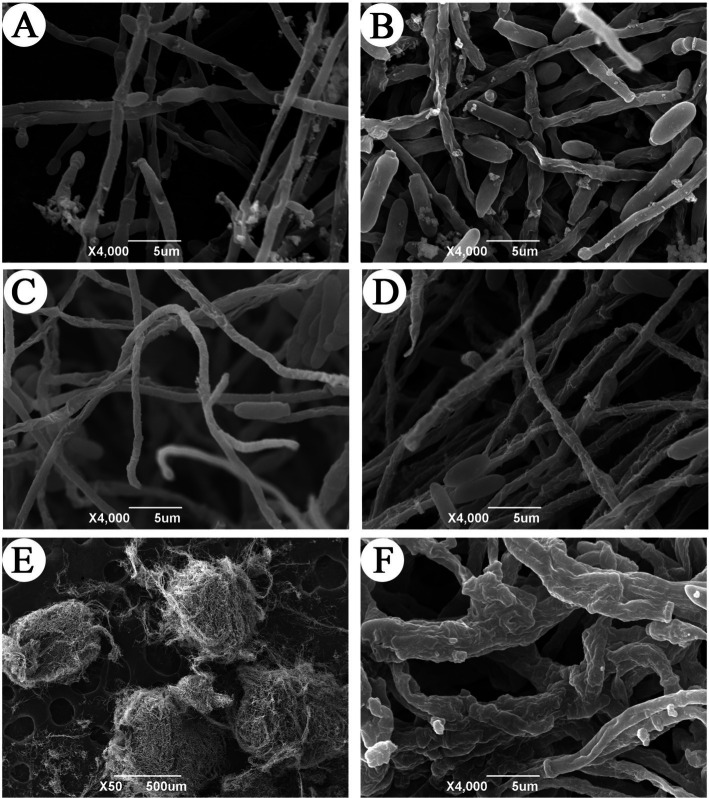
Scanning electron microscope observation of microsclerotia formation in *C. javanica* IF-1106. **(A)** Hyphae in basic medium at 24 h. **(B)** Hyphae in basic medium at 48 h. **(C)** Hyphae in induction media at 24 h. **(D)** Hyphae in the induction media at 48 h. **(E)** Microsclerotia. **(F)** Mycelial structure of microsclerotia surface.

The morphology of hyphae and microsclerotia of *C. javanica* were observed with SEM (Figure 2) in both basic medium and a microsclerotia-inducing medium. The mycelium produced in the microsclerotia-inducting media (Figure 2C) was thinner in diameter than hyphae grown in the basic medium at 24 h (Figure 2A). By 48 h, the surface of the mycelium in basic medium was relatively smooth (Figure 2B), but the surface of the mycelia grown in the induction medium was rough, with a different ultrastructure (Figure 2D). At 48 h, the mycelium in the induction medium was highly entwined, and mature microsclerotium had formed (Figure 2E). In the process of formation of microsclerotia, the mycelia fused and became thicker (Figure 2F).

### 3.2 Surface hydrophobicity of microsclerotia

**Figure 3:**
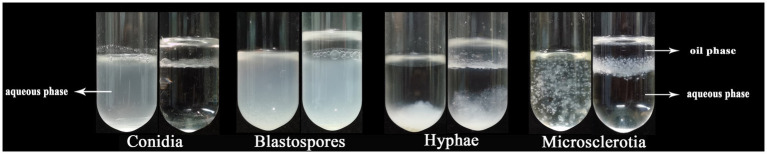
The surface hydrophobicity of different cell types of *Cordyceps javanica* IF-1106.

The surface hydrophobicity of microsclerotia and other *C. javanica* cell types was assessed by the microbial adhesion to hydrocarbons (MATH) assay. In this assay, aerial conidia and microsclerotia collected at the oil–water interface, whereas blastospores and hypha were hydrophilic and were distributed in the aqueous phase (Figure 3).

**Figure 4:**
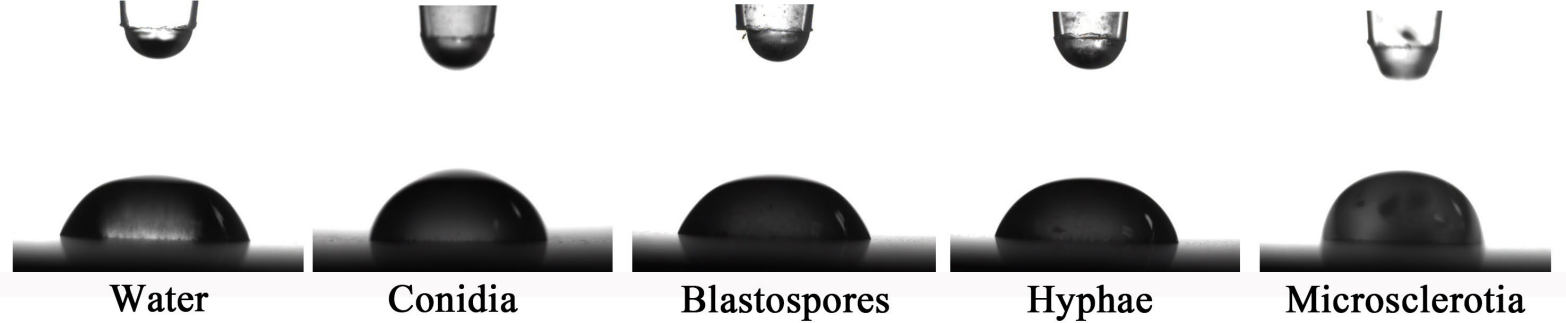
Contact angle measurements. Images are of water droplets resting on various *C. javanica* cell types.

The contact angles of blastospores and droplets of the hyphal suspension were close to that of water. The presence of aerial conidia and microsclerotia obviously increased the contact angle [contact angle: (*F*_(4,49)_ = 47.334, *p* < 0.05); surface tension: (*F*_(4,49)_ = 33.564, *p* < 0.05)], which may have resulted from the cell wall’s physicochemical properties, including its hydrophobicity (Figure 4 and Table 1). Thus, aerial conidia and microsclerotia were the most hydrophobic, while blastospores and hyphae were hydrophilic, which is consistent with our observations of the behavior of these cells in the microbial adhesion to hydrocarbons (MATH) assay (Figure 3).

**Table 1:** Contact angle and surface tension values of the *Cordyceps javanica* cell types.

Cell types	Contact angle	Surface tension
Water	71.45 ± 0.92c	65.57 ± 3.55a
Aerial conidia	76.05 ± 4.09b	60.20 ± 2.00c
Blastospores	69.69 ± 0.92 cd	62.75 ± 2.75b
Hyphae	68.54 ± 0.92d	64.84 ± 2.28ab
Microsclerotia	83.07 ± 2.86a	54.05 ± 1.67d

Data are means ± SD. Data with different lowercase letters indicate significant difference in Duncan’s multiple range tests at *p* < 0.05.

### 3.3 Thermotolerance of *Cordyceps javanica* IF-1106 microsclerotia

**Figure 5:**
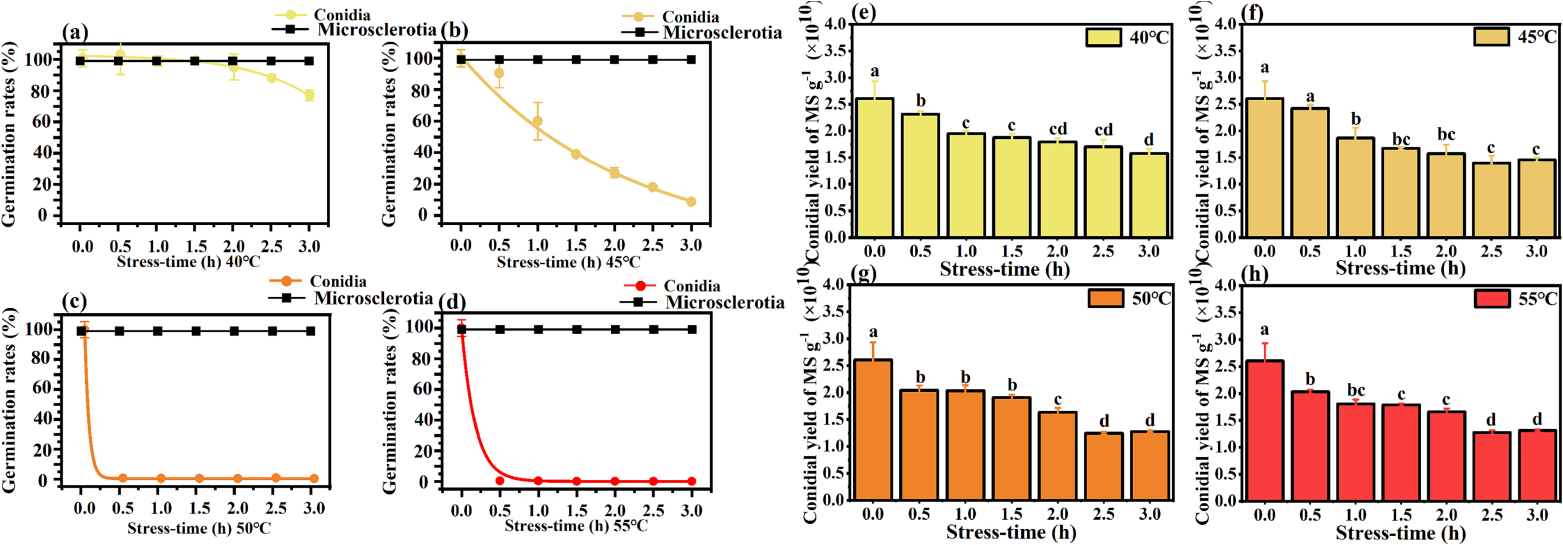
Thermotolerance of germination **(a–d)** and conidial yield **(e–h)** of *Cordyceps javanica* IF-1106 microsclerotia at different temperatures and different stress times. Different lowercase letters indicate significant differences in Duncan’s multiple range tests at *p* = 0.05.

Heat stress from elevated temperatures is a critical environmental constraint for the successful development and efficacy of entomopathogenic fungi (da Paixão et al., 2023). Germination rates of *C. javanica* conidia and microsclerotia after exposure to thermal stress at 40–55°C (Figure 5) showed that microsclerotia were more heat-resistant than conidia. Germination of conidia decreased when the treatment temperature was above 45°C. Exposure of conidia to 55°C led to substantially lower germination 0.75%, in comparison 100% for microsclerotia.

After rehydration, sporulation ability of the heat-treated microsclerotia was evaluated. Though the heat treatment did not affect the germination of microsclerotia, the conidial yield was reduced with increased period of heat stress (Figure 5).

### 3.4 UV-B tolerance of *Cordyceps javanica* IF-1106 microsclerotia

**Figure 6:**
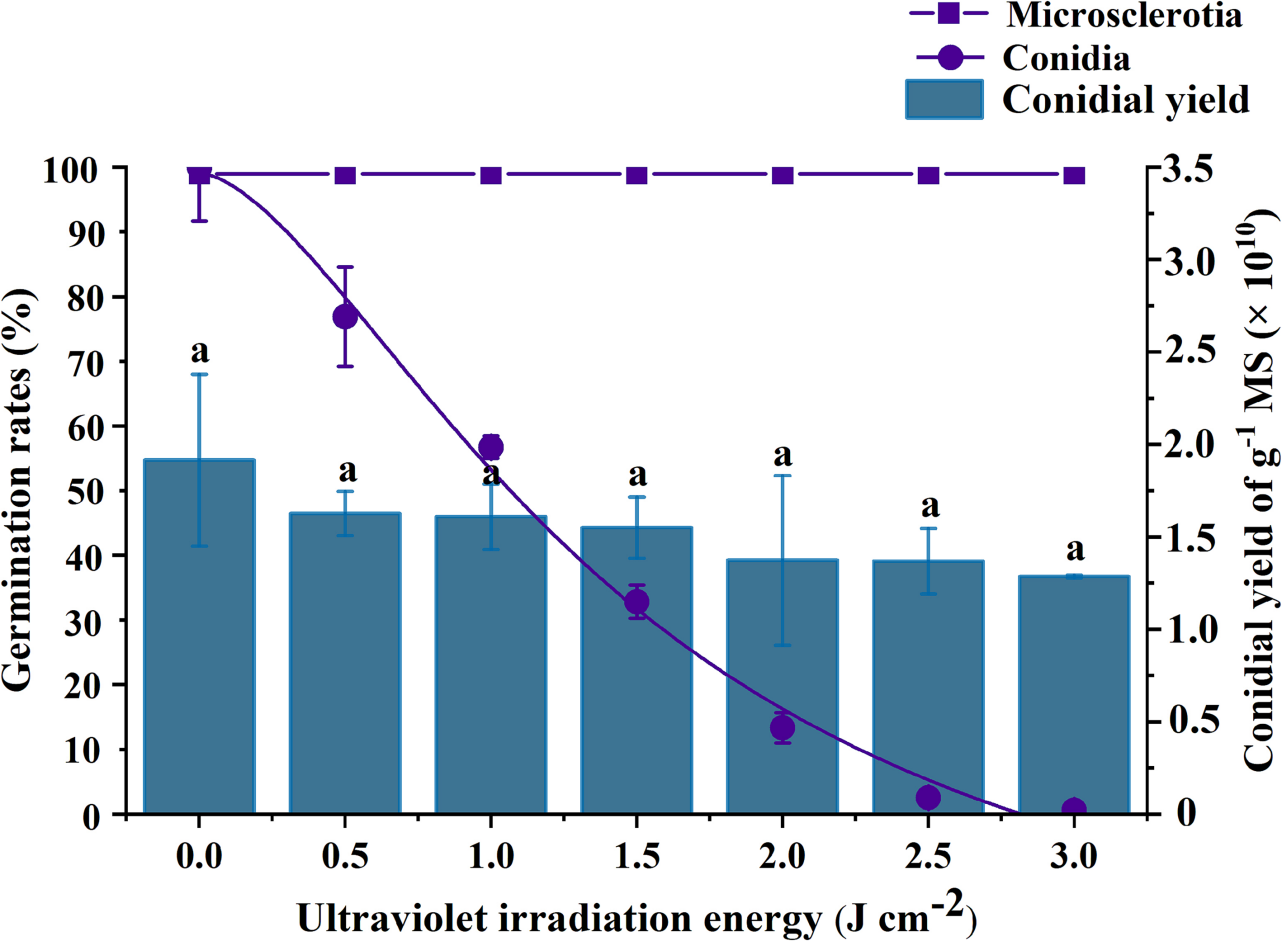
Effect of UV-B radiation exposure on the percentage germination of conidia or microsclerotia and the yield of conidia from microsclerotia of *C. javanica* IF-1106. Different lowercase letters indicate a significant difference in Duncan’s multiple range test at *p* = 0.05.

The rate of germination of microsclerotia was significantly higher than that of conidia under same dosage of UV-B radiation (*p* < 0.05) (Figure 6). Conidia were completely inactivated at 3 J cm^−2^ of UV-B radiation there was no significant effect of UV-B exposure on microsclerotia germination or conidial yield [*F*_(6,11)_ = 1.385; *p* = 0.302], which ranged from 1.29 × 10^10^ to 1.92 × 10^10^ conidia per g of microsclerotia-DE (Figure 6).

### 3.5 Storage stability of *Cordyceps javanica* IF-1106 microsclerotia

**Figure 7:**
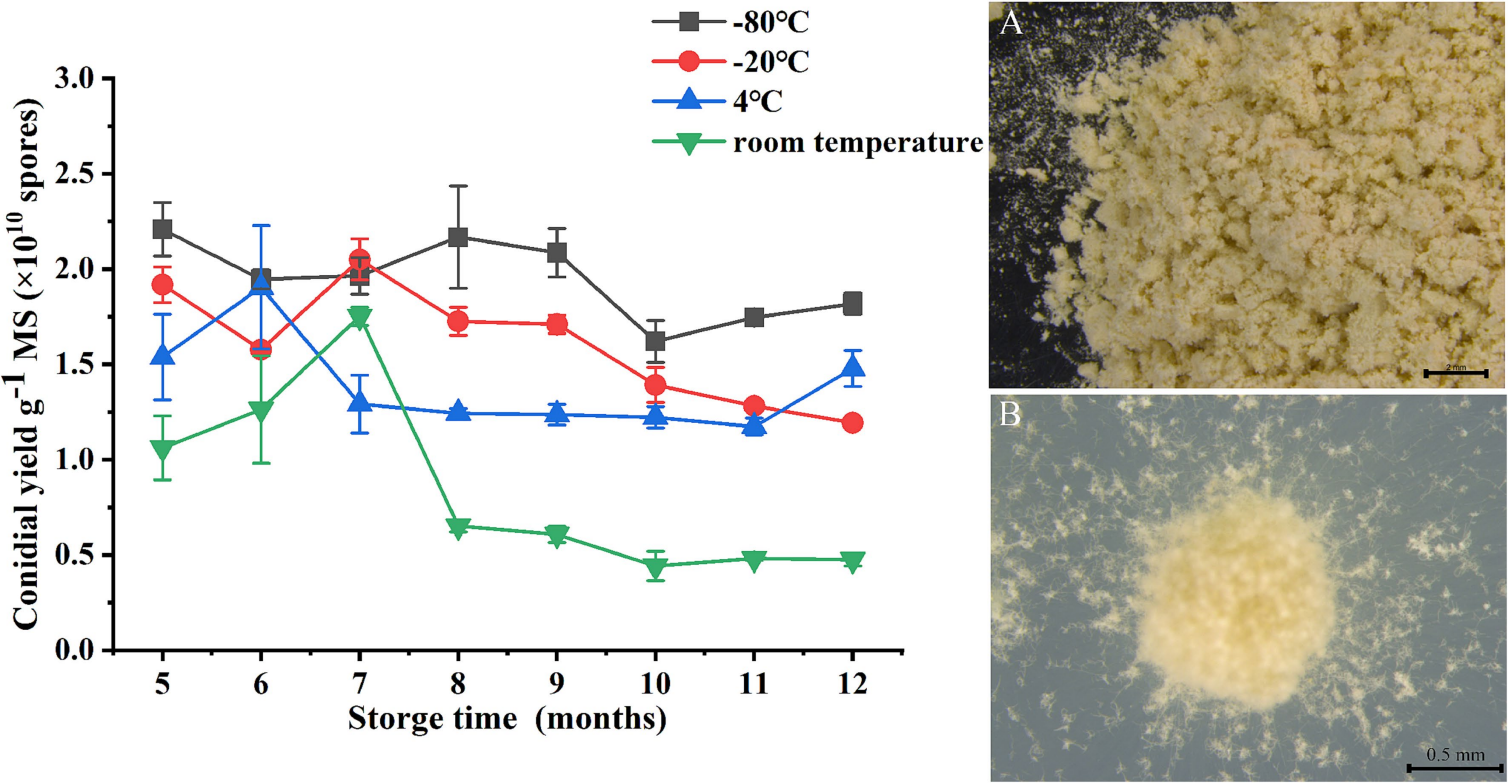
Impact of storage temperature on conidial yield of *C. javanica* IF-1106 microsclerotia. **(A)** Dried microsclerotia-DE. **(B)** Hyphal growth and conidia production from dried microsclerotia on water agar.

When portions of the microsclerotia-DE formulation that had been stored for 5 to 12 months were sprinkled on a water agar plate, hyphal germination occurred after rehydration, leading to formation of aerial conidia on the surface of the microsclerotia and, eventually, mycelia coverage of the whole surface of the agar plate (Figure 7). After 5 months of storage, there was a difference in spore yield of microsclerotia stored at different s temperatures, but spore yield was about 0.44–2.21 × 10^10^ spores g^−1^. Interestingly, between 5 and 7 months of storage, spore yield of microsclerotia (at different storage temperatures) reached its maximum value. Among storage temperatures, spores yield at −80°C was 2.21 × 10^10^ spores g^−1^ at 5 months of storage, 5.5 times that of the yield at the start of storage. After 7 months of storage, the sporulation capacity of microsclerotia decreased but to different extents for different storage temperatures. The conidiogenesis of microsclerotia at room temperature decreased to 0.48 × 10^10^ spores g^−1^, while that of microsclerotia at −80°C remained at 1.82 × 10^10^ spores g^−1^. The spores yield of microsclerotia at 4°C was 1.48 × 10^10^ spores g^−1^ at 12 months of storage.

### 3.6 Ovicidal activity of *Cordyceps javanica* IF-1106 microsclerotia against root-knot nematodes

**Figure 8:**
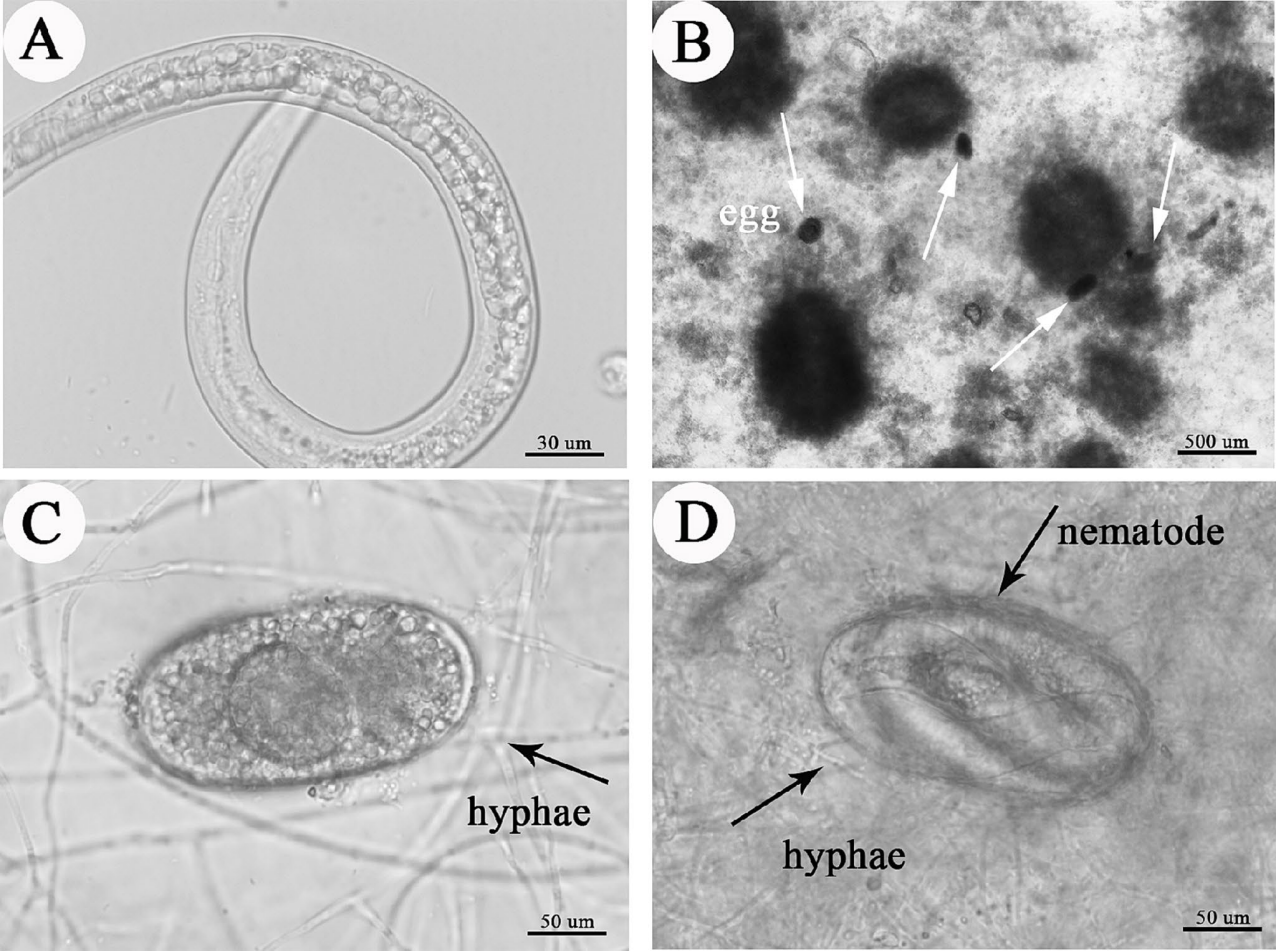
Effects of microsclerotia on root knot nematode eggs, depicting **(A)** hatching nematode; **(B)** microsclerotia and nematode eggs, where white arrows indicate nematode eggs; **(C)** infection of eggs by hyphae from microsclerotia; and **(D)** fungal-infected nematode.

For effective pest control, in addition to remaining viable, the microsclerotia propagules must infect and kill hosts under crop conditions. In our laboratory ovicidal laboratory test, *C. javanica* microsclerotia inhibited development of root-knot nematode eggs (Figure 8 and Table 2). While eggs of RKNs hatched normally in the absence of microsclerotia (Figure 8A), when microsclerotia were present, they produced extensive hyphal networks that trapped nematode eggs (Figures 8B,C). In addition, nematodes that did emerge from eggs were surrounded by the mycelium, preventing them from moving freely (Figure 8D). The rate of infection of root-knot nematode eggs by the fungus increased significantly with increased concentration of the inoculation [*F*_(4,24)_ = 6.718, *p* < 0.05]. The rate at which nematode eggs hatched was reduced to 11.8% by *C. javanica* IF-1106 microsclerotia at a concentration of 2,000 microsclerotia mL^−1^ (Table 2).

**Table 2:** Rates (%) of fungal infection and root knot nematode hatch after treatment by *Cordyceps javanica* IF-1106 microsclerotia at different microsclerotia concentrations.

Microsclerotia concentration (mL^−1^)	Rate of infection (%) by fungus	Nematode hatch rate (%)
0	—	75.5 ± 14.4a
50	26.0 ± 1.7d	70.6 ± 3.3b
100	52.2 ± 7.1c	46.5 ± 7.3c
500	59.7 ± 11.4bc	36.1 ± 11.3c
1,000	74.9 ± 2.3ab	16.5 ± 3.9d
2,000	83.5 ± 4.8a	11.3 ± 6.1e

Data are means ± SD. Different lowercase letters indicate significant differences in Duncan’s multiple range tests at *p* < 0.05.

### 3.7 Control efficacy of *Cordyceps javanica* microsclerotia of root-knot nematodes on cucumber

The pest control of *C. javanica* microsclerotia against root-knot nematode under laboratory conditions was assessed in a trial with greenhouse cucumbers, in terms of root galls control efficiency of root knots (as the percentage reduction in root knots compared to the control) and the growth of cucumber plants. The number of root-knots was significantly reduced by microsclerotia applications compared to an untreated control. Application of *C. javanica* microsclerotia was effective (90.6% reduction) against root-knots of the nematodes at doses as low as 50 microsclerotia 100 g^−1^ soil (Table 3).

**Table 3:** Effects of *C. javanica* IF-1106 microsclerotia agent on root-knot nematode on potted cucumber plants under greenhouse conditions.

Dosage of microsclerotia agent (100 g^−1^ soil)	Number of root-knot per seedling	Control efficiency (%)
0	48.80 ± 7.79a	—
1 × 10^7^	2.60 ± 1.82b	94.7 ± 3.7a
50	4.60 ± 2.07b	90.6 ± 4.3abc
100	5.60 ± 2.70b	88.5 ± 5.5bc
500	7.00 ± 1.73b	85.7 ± 3.6c
1,000	6.40 ± 1.95b	86.9 ± 4.0c
2,000	3.60 ± 1.34b	92.6 ± 2.7ab

Data are means ± SD. Data with different lowercase letters were significant different in Duncan’s multiple range tests at *p* < 0.05. The dosage of 1 × 10^7^ as control (fungal conidia).

**Figure 9:**
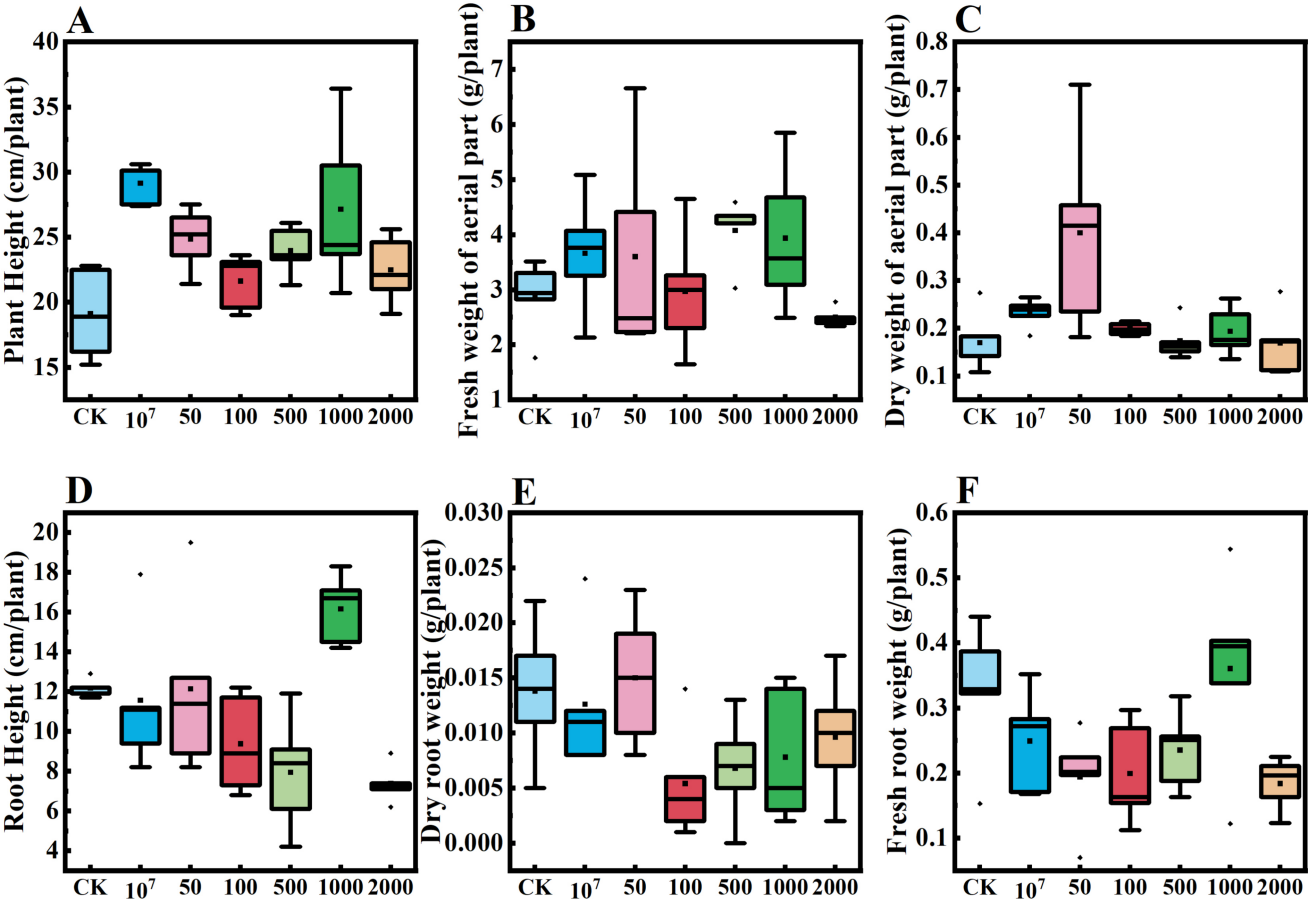
Effect of dosages of *Cordyceps javanica* IF-1106 microsclerotia on growth of cucumber plants in a greenhouse trial. **(A–F)** Plant height, fresh weight of aerial part, dry weight of aerial part, root weight, underground dry weight, underground fresh weight of the cucumber plants.

Results from our potted cucumber trial showed that the growth of the crop was significantly enhanced by the application of the microsclerotia preparation of *C. javanica* IF-1106 (Figure 9, *p* < 0.05). The plant height, fresh weight, and dry weight above ground were all significantly higher than the control (Figures 9A–C). When the fungal dosage was increased to 2,000 microsclerotia 100 g^−1^ soil, there was no obvious promotion effect on cucumber growth (Supplementary Figure S1). The root height, underground fresh weight, and dry weight were lower at this high dosage than in the control (Figures 9D–F). In conclusion, microsclerotia provided control of the root-knot nematode and reduced the number of galls on cucumber roots.

## 4 Discussion

Liquid fermentation of the bio-insecticidal fungus *C. javanica* in specific medium supports the formation of microsclerotia. The formation cycle and morphological structure are different among species and strains (Villamizar et al., 2018). After 4 days of culture, *Beauveria bassiana* showed a strong aggregation of mycelia, and mature microsclerotia were formed after 10 days. However, hyphal aggregation and the formation of microsclerotia in *C. javanica* were observed in 48 h and 3 days, respectively. This difference between species may be related to the species, its geographical origin, or the culture conditions of the species. Three pathways have been recognized that can lead to microsclerotia formation in entomopathogenic fungi: coagulative, non-coagulative, and hyphal element agglomeration (Supplementary Figure S2 and Table 4). In the coagulative type, spores aggregate before germination, but then rapidly germinate. Hyphal tip growth (Zhang and Zhang, 2016) leads to further aggregation, resulting ultimately in pellet formation (Supplementary Figure S2A). In contrast, spores of the non-coagulative type germinate before aggregation or pellet formation. Therefore, one pellet theoretically could be formed by a single spore (Supplementary Figure S2B). It should be noted, however, depending on cultivation factors, fungal species will exhibit different morphological behavior. In our study, the conidia of *C. javanica* germinated to hyphal elements firstly, then the hyphal elements agglomerated, leading to the formation of pellets (Supplementary Figure S2C).

**Table 4:** Fungal species exhibiting the aggregation types.

Pellets type	Fungal species	Reference
Coagulative	*A. niger*	Zhang and Zhang (2016)
	*A. nidulans*	
	*A. niger*	
	*A. oryzae*	
	*P. chrysosporium*	
Non-coagulative	*Rhizopus* spp.	
	*Mucor* spp.	
	*P. chrysogenum*	
	*Mortierella vinacea*	
	*A. ochraceus* ATCC 3150	
	*Rhizopus oryzae*	

The surface properties of fungal cells are the basis for the host-fungus interaction. For example, greater cell surface hydrophobicity is associated with increased virulence in *Candida dubliniensis* strains, and the hydrophobic rodlet layer of conidia also appears to protect the fungus against the host immune reactions (Hazen, 2004). In *Aspergillus* spores, the rodlet layer of the spores and its hydrophobin constituents contribute to hydrophobicity, adhesion to the host, and protects fungal cells from the host’s alveolar macrophages (Holder et al., 2007). Hydrophobic entomopathogenic fungal spores, of species such as *Nomuraea rileyi*, *Metarhizium anisopliae* and *Paecilomyces fumosoroseus*, possessed well defined outer rodlet layers. In comparison, hydrophilic fungi, such as *Hirsutella thompsonii* and *Verticillium lecanii*, are lack of a rodlet layer but have an outer mucilaginous coat that is produced at spore maturation (Boucias et al., 1988).

For *C. javanica* IF-1106, we found that different stages, such as aerial conidia, blastospores, hypha and microsclerotia, had distinct surface features that affected their hydrophobicity. Our data suggest that *C. javanica* IF-1106 produces several distinctly different infectious cell types, which allows this strain to interact with a variety of host surfaces, and this cellular diversity may help account for the fungus’ broad invertebrate host range.

The development of microbial biocontrol agents depends in part on the ability of microorganisms to form stable, infective propagules. However, environmental factors such as high temperatures and solar UV radiation can harm these fungi and thereby limit their ability to control pests (Kobori et al., 2015). While past work with entomopathogenic fungi have mostly focused on use of conidia as the infective cell type, studies have found that the germination rate in *B. bassiana* of microsclerotia is significantly higher than that of conidia under comparable conditions (Yin and Wang, 2022). We found that in *C. javanica* IF-1106 microsclerotia were capable of surviving exposures to high temperature or UV-B radiation and remain able to produce conidia after germination of the microsclerotia. High thermotolerance and UV resistance after application is favorable for successful use in pest control of such fungi, especially under host conditions exposed to sunlight.

Fungal biocontrol agents intended for use as biopesticides must survive the drying processes during production and formulation and have good product shelf life at refrigerated or, preferably, at room temperature (Kobori et al., 2015). The *Beauveria pseudobassiana* isolate AgR-F704 exhibited stability at 4°C and at 20°C (100 and 68% germination, respectively) after 6 months of storage. The microsclerotia of *C. javanica* retained viability even after 12 months of storage at room temperature, 4, −20, or −80°C, with a 100% germination rate, which was much higher than that of the conidia of *C. javanica*, when formulated in an oil suspension or of as sporangium powder (Wang, 2022). Although the yield of conidia from microsclerotia decreased significantly after 1 year of storage at room temperature, a 4 × 10^9^ conidial yield g^−1^ microsclerotia-DE was still obtained. It is possible that the apparently lower rate of germination after storage was due to an induced dormancy state or a reduction in microsclerotia vigor as a result of the long-term storage conditions. After storage for 1 year under refrigerated conditions, all microsclerotia germinated and spores yield >1.19 × 10^10^ spores g^−1^. Our current study showed that air-dried microsclerotia of *C. javanica*, with a low moisture content (3–5%), exhibited good stability for >1 year.

Entomopathogenic fungi can be an effective method to control plant parasitic nematodes that live in soil (Kepenekci et al., 2017). A pot experiment under greenhouse conditions using microsclerotia of *Paecilomyces lilacinus* against the root galling nematode *Meloidogyne incognita* reduced galls by 88.2% and eggs by 76.9% egg mass reduction (Khalil et al., 2012). In our study, *C. javanica* IF-1106 microsclerotia provided up to 92.6% reductions in galls of RKNs. In addition, the dose and application method had a large influence on the level of biocontrol of *C. javanica* IF-1106 achieved by application of microsclerotium. Our results found the best control at a relative low dose of *C. javanica* IF-1106 microsclerotia and excessively high concentrations of microsclerotia had negative effects on plant growth. Similar results were found by Kepenekci et al. (2017), in which a 10^8^ CFU mL^−1^ of *B. bassiana* showed the highest level of control (98.6% inhibition of *Meloidogyne hapla*) but also found that much higher levels (10^6^ CFU mL^−1^) reduced tomato plant growth. Overall, in our study, application of *C. javanica* microsclerotia had promising activity for control of root-knot nematodes, as well a positive effect on cucumber growth.

Microsclerotia are a dormant structure that needs to form a mycelium and produce spores that germinate into to infect a host, such as nematode eggs. When mycelia occur at low densities, they can only entrap nearby eggs, but not distant ones. Therefore, it is likely that microsclerotia applications targeting root knot nematodes will have to be made in advance of damaging nematode infestations to allow the fungus time to colonize the plant rhizosphere. Then, when field infestations of root knot nematodes develop, the fungal hyphae and conidial spores will be present, allowing them to immediately being infecting nematode eggs.

At present, research and development on microsclerotia preparations for biological control are still in the laboratory stage. Although microsclerotia can be produced by liquid culture, the overall level is low (the yield is about 10^4^ microsclerotia mL^−1^) compared to conidia (10^8^–10^9^ CFU mL^−1^). Because the yield of microsclerotia is relatively low per unit of medium, their production cost is significantly higher than that of conidia. Optimizing rearing conditions and improving the fermentation level for microsclerotia are key to large-scale production and application. In addition, the development of appropriate application technology is critical for successful commercialization of microsclerotial preparations. Microsclerotia can be formulated as particles or granules, and treatment may be made during soil mixing (in greenhouse crops), via injection application, or through root irrigation. Also, microsclerotia can be combined with other biocontrol products or low-toxic chemicals to more effectively prevent or control soil-transmitted pathogens.

In summary, *C. javanica* IF-1106 was able to produce microsclerotia under specific induction conditions in liquid culture. The microsclerotia were more tolerant to desiccation and also exhibited higher heat and UV-B stress resistance and storage stability. When the microsclerotia of *C. javanica* IF-1106 were applied to soil infested by nematodes they produced infective hyphae and conidia that suppressed hatching of root-knot nematode eggs and promoted cucumber growth. All these properties suggest that the *C. javanica* IF-1106 microsclerotia propagules can be likely be developed further as a mycoinsecticide.

## Data Availability

The original contributions presented in the study are included in the article/Supplementary material, further inquiries can be directed to the corresponding authors.
