# Supplementary material for: Microsclerotia formation of the biocontrol fungus Cordyceps javanica IF-1106 and evaluation of its stress tolerance and pathogenicity
Source: Front Microbiol. 2025 Apr 29;16:1583850. doi: 10.3389/fmicb.2025.1583850 (PMC12069389; doi:10.3389/fmicb.2025.1583850)
Supplement: Supplementary file 1 [file Data_Sheet_1.docx]

***Supplementary Material***

**Microsclerotia formation of the biocontrol fungus** ***Cordyceps javanica* IF-1106 and evaluation of its stress tolerance and pathogenicity**

**Yihua Li, Junmei Li, Xiaoxia Cai, Meiyu Gao, Hongliang Diao, Huiming Xiang, Wenwen Zhou^*^, Ruiyan Ma^*^**

*** Correspondence:** College of Plant Protection, Shanxi Agricultural University, Taigu, Shanxi 030801, PR China. E-mail: wenwenzhoumail@163.com (Wen-wen Zhou); mary@sxau.edu.cn (Rui-Yan Ma)


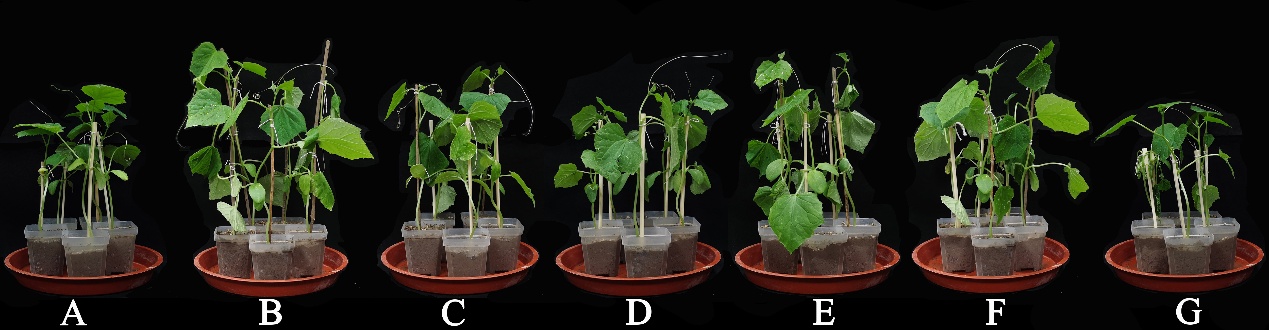


**Figure S1** Effect of dosages of *C. javanica* IF-1106 microsclerotia agent on growth of cucumber: (A) plant not inoculated with microsclerotia; (B) 1×10^7^ conidial spores mL^-1^; (C-G) 50, 100, 500, 1000, and 2000 microsclerotia 100 g^-1^ soil.


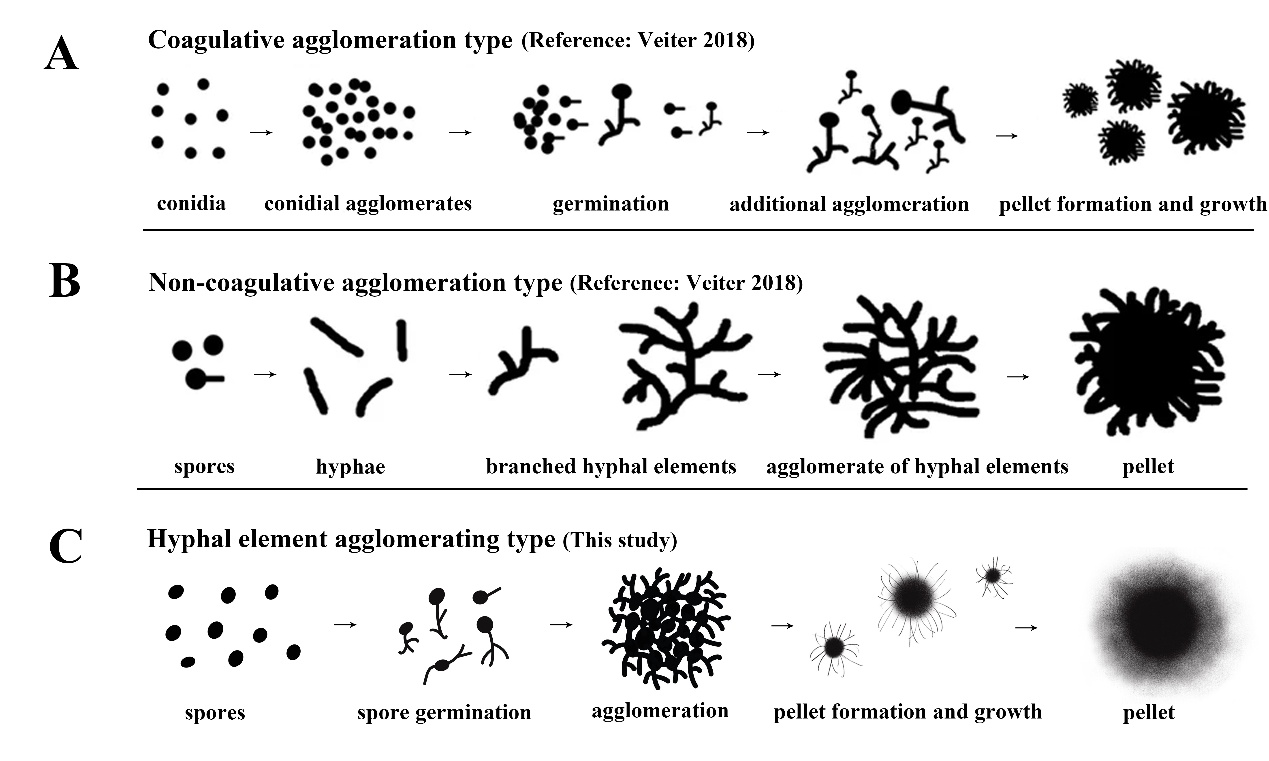


**Figure S2** Diagram showing general pathways for microscerotia formation in different groups of fungi
